# Type-1 interferons contribute to oxygen glucose deprivation induced neuro-inflammation in BE(2)M17 human neuroblastoma cells

**DOI:** 10.1186/1742-2094-11-43

**Published:** 2014-03-06

**Authors:** Myles Robert Minter, Moses Zhang, Robert Charles Ates, Juliet Marie Taylor, Peter John Crack

**Affiliations:** 1 Department of Pharmacology, University of Melbourne, 8th floor, Medical building, Grattan St, Parkville 3010, VIC, Australia

**Keywords:** Type-1 interferon, Neuro-inflammation, Hypoxia-ischaemia, Cytokines, JAK-Stat

## Abstract

**Background:**

Hypoxic-ischaemic injuries such as stroke and traumatic brain injury exhibit features of a distinct neuro-inflammatory response in the hours and days post-injury. Microglial activation, elevated pro-inflammatory cytokines and macrophage infiltration contribute to core tissue damage and contribute to secondary injury within a region termed the penumbra. Type-1 interferons (IFNs) are a super-family of pleiotropic cytokines that regulate pro-inflammatory gene transcription via the classical Jak/Stat pathway; however their role in hypoxia-ischaemia and central nervous system neuro-inflammation remains unknown. Using an *in vitro* approach, this study investigated the role of type-1 IFN signalling in an inflammatory setting induced by oxygen glucose deprivation (OGD).

**Methods:**

Human BE(2)M17 neuroblastoma cells or cells expressing a type-1 interferon-α receptor 1 (IFNAR1) shRNA or negative control shRNA knockdown construct were subjected to 4.5 h OGD and a time-course reperfusion period (0 to 24 h). Q-PCR was used to evaluate IFNα, IFNβ, IL-1β, IL-6 and TNF-α cytokine expression levels. Phosphorylation of signal transducers and activators of transcription (STAT)-1, STAT-3 and cleavage of caspase-3 was detected by western blot analysis. Post-OGD cellular viability was measured using a MTT assay.

**Results:**

Elevated IFNα and IFNβ expression was detected during reperfusion post-OGD in parental M17 cells. This correlated with enhanced phosphorylation of STAT-1, a downstream type-1 IFN signalling mediator. Significantly, ablation of type-1 IFN signalling, through IFNAR1 knockdown, reduced IFNα, IFNβ, IL-6 and TNF-α expression in response to OGD. In addition, MTT assay confirmed the IFNAR1 knockdown cells were protected against OGD compared to negative control cells with reduced pro-apoptotic cleaved caspase-3 levels.

**Conclusions:**

This study confirms a role for type-1 IFN signalling in the neuro-inflammatory response following OGD *in vitro* and suggests its modulation through therapeutic blockade of IFNAR1 may be beneficial in reducing hypoxia-induced neuro-inflammation.

## Background

Hypoxic-ischaemic brain injury, including stroke and traumatic brain injury (TBI), are the most common acute central nervous system (CNS) neuro-degenerative disorders worldwide. Pathologically these injuries begin with an initial tissue insult and reduction in oxygen and glucose levels in the surrounding extracellular space, developing a core infarct. A secondary injury also ensues known as the penumbra, where neighbouring cells are exposed to the microenvironment generated by the core infarct. The development of the penumbra is dependent on a host of physiological processes. Hallmarks of neuro-inflammation including activated microglia [1], astrogliosis [2], and increased cytokine and chemokine levels [3] have all been reported in animal models and postmortem human brain samples of stroke and TBI pathology. In cases of severe trauma where blood-brain barrier disruption is evident, peripheral immune cells, namely macrophages and neutrophils, have been reported to infiltrate the injury site and perpetuate an inflammatory response [4,5]. In stroke pathology, matrix metalloproteinases are released and compromise blood brain barrier integrity [6]. Within hours of stroke incidence, neutrophils and macrophages are detected crossing the permeated barrier into the ischemic hemisphere [7]. Initially a neuro-inflammatory response is beneficial in the clearance of cellular debris and foreign matter, however, failure to resolve this inflammation results in greater infarct sizes and poorer prognosis [8]. The generation of pro-inflammatory mediators within the CNS occurs through multiple cell types and contributes to acute neuro-degeneration. Fragmented DNA/mRNA, heat shock proteins and hyaluronic acid released from necrotic cells act as ligands for Toll-like receptors (family of pattern recognition receptors) located in high density on resident microglia [9-11]. Upon activation, microglia secrete pro-inflammatory cytokines including IL-1β, IL-6 and TNF-α [12,13] and this inflammatory milieu triggers necrosis, apoptosis, necroptosis and excitotoxicity. The chemokines (C-C motif) ligand-2 (CCL-2) and CCL-3, also released by microglia, lead to recruitment of peripheral macrophages and neutrophils [14] driving a self-perpetuating, neuro-degenerative cytokine storm. Though microglia are the predominant cell-type responsible for the development of a CNS inflammatory response, neurons themselves also contribute to this pro-inflammatory environment. Brain-localised neuronal populations express TLRs [15,16] and can respond to damage associated molecular patterns (DAMPs) released in hypoxia-ischaemia injuries. Downstream signalling upon DAMP recognition involves modulation of neuronal IL-1β, IL-6 and TNFα secretion in excitotoxic conditions [17]. Clearly, neurons not only respond to neuro-inflammation but also secrete pro-inflammatory cytokines themselves. In a neuro-inflammatory setting, microglia and neurons act in a symbiotic fashion allowing a cytokine storm to manifest. In the periphery, type-1 interferons (IFNs) are key regulators of this cytokine storm [18,19]; however their role in CNS neuro-inflammation and hypoxic-ischaemic injury is still not well understood.

Interferons (IFNs) are a super-family of pleiotropic cytokines that play a pivotal role in host immune response to infections, pathogens and various diseases [20]. The super-family consists of type-1 (IFNα and IFNβ), type-2 (IFNγ) and type-3 (IFNλ) interferon subtypes which all possess vastly different functions in innate immunity. IFNγ is critical in controlling glial phenotype [21] and peripheral haematopoetic cell infiltration [22] following *in vivo* hypoxia-ischaemia insult, which contributes to the developing penumbra. The roles of type-2 and type-3 IFNs are not addressed here, rather, this study focuses on the type-1 IFNs in a neuronal context. Type-1 IFNs display pro-inflammatory properties via activation of multiple signalling cascades, heavily dependent on cell type, including the classical JAK/STAT pathway. Specifically, type-1 IFNs can induce pro-inflammatory gene transcription leading to the secretion of cytokines (including TNF-α, IL-6 and IL-1β), cellular recruitment and inflammatory progression. Indeed their production is not limited to the periphery with CNS neurons and microglia known to secrete [23,24] and respond [25] to type-1 IFNs. Elevated type-1 IFN levels have been reported in various neuro-pathologies including: systemic lupus erythematosus [26], HIV-encephalopathy [27], West Nile virus ‘sickness’ [28] and Aicardi-Goutieres syndrome [29,30]. IFN-α transgenic mice display severe neuro-degeneration and cognitive decline [31]. However, the complexities of type-1 production and signalling in the myriad of cell types within the CNS are still not clearly understood.

Critically, type-1 IFNs are involved in the initiation and/or regulation of pro-inflammatory cytokines [18,19] and in their absence a robust innate immune response cannot develop. We hypothesised that type-1 IFNs were therefore critical to the pro-inflammatory cellular response following hypoxia-ischaemia insult contributing to the resulting neuro-degeneration. In this study we used an *in vitro* approach to model hypoxic-ischemic injury and characterise a role for type-1 IFNs. We utilised the human BE(2)M17 neuroblastoma cell line, which expresses mature neuro-filaments, physiologically relevant levels of tyrosine hydroxylase and dopamine-β-hydroxylase and conduct neurotransmission [32]. These properties confer a dopaminergic neuron-like phenotype in a human cell line, which can be easily genetically manipulated, making them a viable candidate to study the effects of OGD. Using these cultures we identified increased type-1 IFN production and signalling in response to hypoxic insult, deleterious to injury outcome in the neuro-inflammatory environment.

## Methods

### Antibodies

Primary antibodies used for western blot analysis: rabbit anti-p-Stat-1 (1:1,000, Cell signalling, 9171S), rabbit anti-p-Stat-3 (1:1,000, Cell signalling, 9145S), rabbit anti-cleaved caspase-3 (1:1,000, Cell signalling, 9661S), rabbit anti-caspase-3 (1:1,000, Cell signalling, 9665S), mouse anti-β-tubulin (1:20,000, Millipore, MAB3408). Secondary antibodies used for western blot analysis: horseradish peroxidise (HRP) conjugated goat anti-rabbit and goat anti-mouse (1:1,000, Dako, P0448 and P0447).

### M17 neuroblastoma cells

Human BE(2)M17 neuroblastoma cells (ATCC® number:CRL-2267™) were cultured in T75 flasks with culture medium (OptiMEM (Gibco), 5% FBS, 0.5% Penicillin-Streptomycin (Gibco)) at 37°C/5% CO_2_ until 90% confluent. Cells were then plated at 1.5 × 10^6^ cells/10 cm dish or 7.5 × 10^4^ cells/24-well plate and incubated for a further 24 h. Cultures were then serum starved for 12 h prior to treatment.

### Generation of M17 IFNAR1 overexpressing cells

Transient IFNAR1 overexpressing M17 cells were generated using a customised IFNAR1 mRNA vector expression system. An optimised nucleotide sequence encoding IFNAR1 mRNA (GenBank: NM_010508) was cloned into a pUC57 vector (GenScript). AttB1 and AttB2 motifs were then ligated to the IFNAR1 mRNA sequence using PCR for use in the Gateway® cloning system (Invitrogen). The AttB1-IFNAR1-AttB2 PCR product was isolated using QIAquick® PCR purification kits (QIAGEN) after 2% agarose gel electrophoresis and sequenced. The purified AttB1-IFNAR1-AttB2 PCR product was then translocated into vector pDONR201 (Invitrogen) using the BP Clonase^©^ II enzyme kit (Invitrogen), according to manufacturer’s guidelines. Upon DH5α bacterial competent cell expansion (kanamycin selection, 50 μg/mL (Gibco)) and plasmid purification, pDONR-IFNAR1 was obtained. The IFNAR1 mRNA sequence was removed from the pDONR201-IFNAR1 plasmid and translocated into a pcDNA6.2/cEM-GFP destination vector using the LR clonase® II kit (Invitrogen) as per manufacturer’s protocol. This reaction yields pcDNA6.2/cEM-GFP-IFNAR1 which upon expansion, purification and sequencing was transiently transfected into parental M17 cells using Fugene®HD (Promega). Sixty to seventy-five percent transfection efficiency was routinely observed by visual assessment of GFP positive cells using a Leica DMI 6000B fluorescence microscope. Fifty-five percent over-expression of IFNAR1 was confirmed using Q-PCR.

### Generation of M17 IFNAR1 knockdown cell line

IFNAR1 (IFNAR1 KD) or negative control (NC) knockdown M17 cells were generated using commercially available shRNA plasmid constructs (Origene). Briefly, M17 cells were transfected using Fugene®HD (Promega) with shRNA plasmids containing an IFNAR1 specific shRNA cassette or non-effective 29-mer scrambled shRNA cassette with a GFP tag. Clonal cell lines were generated using the selectable marker puromycin (0.5 mg/mL, Gibco) and maintained in OptiMEM containing 10% FBS and 1% penicillin-streptomycin. Successful knockdown of IFNAR1 expression was confirmed by Q-PCR where IFNAR1 levels in IFNAR1 KD M17 cells were 80% lower compared to NC M17 cells.

### Interferon α/β treatment

Human IFNα and IFNβ was sourced commercially (PBL Interferon source, 11200-2 and 11415-1) and dissolved in a 0.5% BSA/PBS vehicle. Serum starved M17 cell cultures were treated with IFNα, IFNβ (1,000 U/mL) or vehicle for up to 30 min in fresh culture medium.

### Oxygen glucose deprivation

Serum starved M17 cells were supplemented with DMEM containing no glucose (Gibco) and gassed with nitrogen (95% N_2_, 5% CO_2_) in a 60% humidified air-tight chamber for 5 min. This process of OGD routinely achieved O_2_ levels of 0% to 1% within the chamber. Cultures were incubated at 37°C within the hypoxic chamber for 4.5 h before being released and supplemented with high glucose DMEM to model reperfusion for up to 24 h.

### Protein extraction

Cells were scraped in ice cold PBS and centrifuged at 5,000 × g for 5 min. The resulting pellet was then sonicated in lysis buffer (50 mM Tris, 1% Triton x-100, 1% SDS, PhosphoSTOP® and protease inhibitors (Roche), pH 7.4) and protein concentrations were determined using a Bradford assay (Biorad).

### Western blot analysis

Fifty micrograms of protein extract with 2x Novex® Tris-glycine SDS sample buffer (Invitrogen) was resolved on 10% acrylamide SDS PAGE gels and transferred to polyvinylidene fluoride (PVDF) membranes by semi-dry transfer. Membranes were blocked with 5% w/v skim milk in TBS-T for 1 h before overnight incubation with primary antibodies in 2% w/v skim milk in TBS-T at 4°C. Membranes were then thoroughly washed with fresh TBS-T prior to being incubated with HRP-conjugated secondary antibodies (diluted in 2% skim milk in TBS-T) for 90 min at room temperature. After three further TBS-T washes, signals were detected using an ECL prime® western blotting detection kit (Amersham) and visualised using the IQ350 imaging machine (GE Healthcare). All western blots were technically repeated twice to confirm initial results. Post-image densitometry was performed using Image J software (NIH), whereby signal intensity was calculated in arbitrary units. For densitometry calculations, phosphorylation intensity was measured in arbitrary units and normalised to the β-tubulin loading control. These values were then calculated as fold change compared to a vehicle control. No ANOVA and post-hoc tests could be performed as a result of this normalisation technique.

### RNA isolation and cDNA synthesis

Cells were scraped and lysed in TRIzol® (Invitrogen) with RNA extracted as per manufacturer’s instructions. RNA yield and purity was determined by the Nanodrop 1000 spectrophotometer (Thermo-scientific). One microgram RNA was converted to cDNA using a high capacity cDNA reverse transcription kit (Applied Biosystems) according to manufacturer’s guidelines. Reverse transcription was performed under the following conditions: 25°C for 10 min, 37°C for 120 min then 85°C for 5 min to terminate the reaction. The cDNA product was diluted 1:3 in DEPC-treated H_2_O for use in Q-PCR.

### Quantitative polymerase chain reaction (Q-PCR)

All Q-PCR reactions were performed in standard 384-well plates using the 7,900 ht fast real-time PCR system (Applied Biosystems). Commercially available Taqman probes (Applied Biosystems) were used to analyse huIFNα (Hs00256882_sl), huIFNβ (Hs01077958_sl), huIFNAR1 (Hs01066115_m1), huIL-1β (Hs00174097_m1), huIL-6 (Hs00985639_m1), huTNF-α (Hs00174128_m1) and 18 s rRNA (4352930E) under the following cycle conditions: 50°C for 2 min, 94.5°C for 10 min (97°C for 30 s, 59.7°C for 1 min) × 40 repeats. Fold change in mRNA levels were then calculated using the ΔΔct method (2^-ΔΔct^) relative to no OGD control samples. For all Q-PCR plates, samples were run in triplicate to eliminate potential errors and variance between wells.

### MTT assay

Cell viability was measured by the cellular ability to metabolise 3-(4,5-Dimethylthiazol-2-yl)-2,5-diphenyltetrazolium bromide (MTT) (Sigma) to an insoluble purple formazan product as described previously [33]. All MTT assays were performed in a 24-well format and conducted in triplicate. Following OGD, MTT reagent (80 μg/mL) was added to the cells for 2 h at 37°C. Media was carefully removed and 200 μL DMSO was added to each well to solubilise the precipitated formazan product. This solution was then transferred to a fresh 96-well plate and absorbance was determined at 595 nm. Viability of treated samples was expressed as a percentage of Abs_595nm_ of the no OGD control. For all MTT assays, cells were plated in triplicate to eliminate potential errors due to differences in cell density or inter-well absorbance.

### Statistical analysis

In all MTT assay data an unpaired two-tailed student’s *t*-test was used to compare non-transfected M17 and IFNAR1 overexpression M17 groups, or M17 NC shRNA and M17 IFNAR1 KD groups where *P* <0.05 was considered significant. An unmatched one or two-way ANOVA was performed, where suitable, for the Q-PCR and densitometry data for each time-point with cellular genotype or IFN subtype as the fixed variable. A Bonferroni or Dunnett’s post-hoc test was then performed to determine significance (*P* <0.05). Graphical data are represented as mean ± SEM where each independent experiment is represented by ‘n’. All statistical analysis was conducted using PRISM® 5 (GraphPad).

## Results

### Type-1 IFN-dependent phosphorylation of stat isoforms is subtype specific

**Figure 1:**
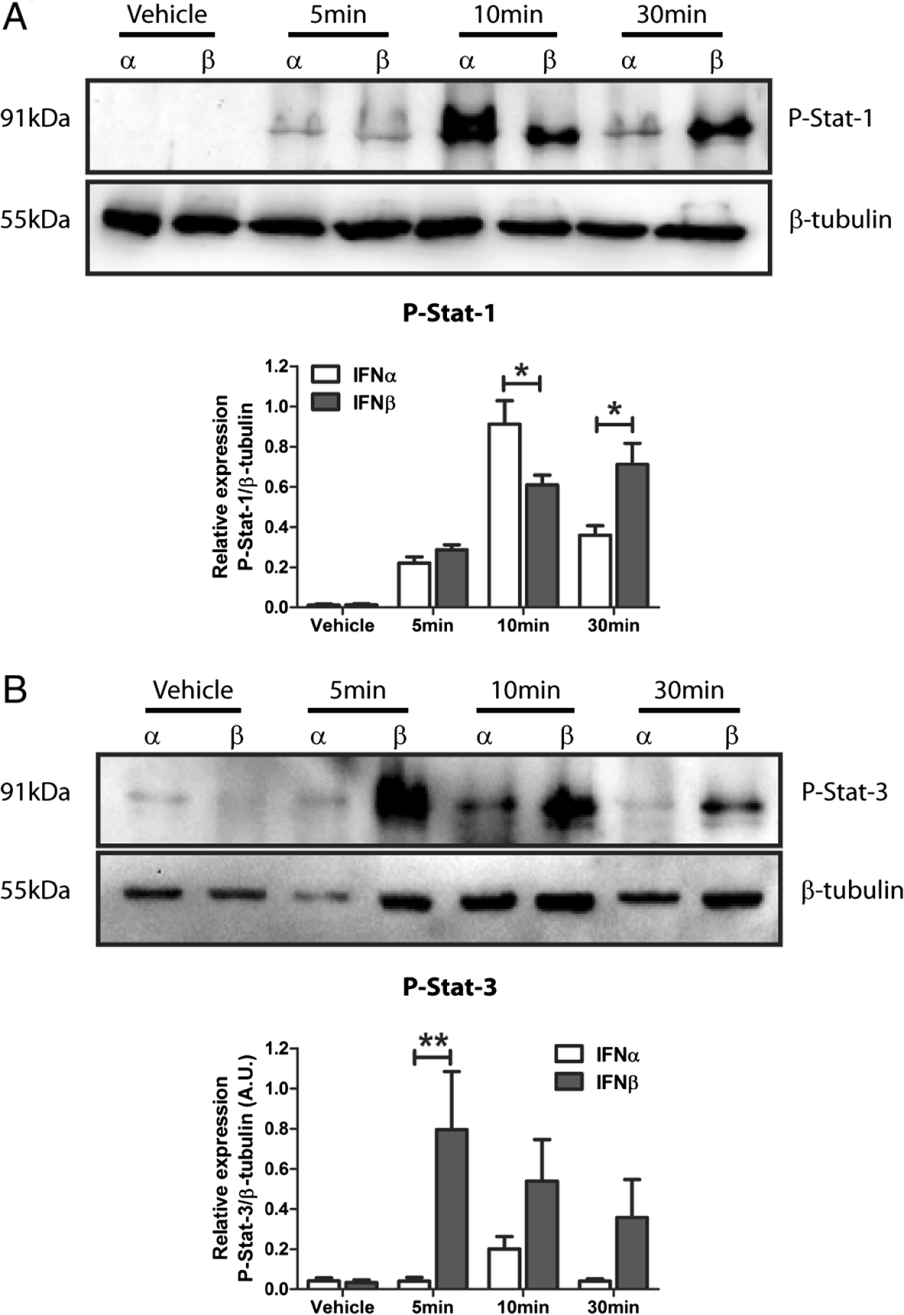
**Type-1 interferon α and β induce differential phosphorylation of Stat isoforms. (A)** Representative western blot of IFNα/β (1,000 U/mL) treated M17 cells probed for P-Stat-1 and corresponding densitometry (*P <0.05, n = 3, unmatched two-way ANOVA, Bonferroni post-hoc test). **(B)** Representative western blot of IFNα/β (1,000 U/mL) treated M17 cells probed for P-Stat-3 and corresponding densitometry (**P <0.01, n = 3, unmatched two-way ANOVA, Bonferroni post-hoc test). For densitometry calculations, phosphorylation intensity was measured in arbitrary units (A.U.) and normalised to the β-tubulin loading control. Graphical data are represented as mean ± SEM.

Activation of the Jak-Stat signalling cascade leads to robust pro-inflammatory cytokine secretion and NF-κB phosphorylation, hallmarks of classical neuro-inflammation. Type-1 IFNs activate the Jak-Stat cascade, thus we investigated the ability of type-1 IFNs to phosphorylate Stat isoforms in M17 human neuroblastoma cells by western blot analysis. Cells treated with either IFNα and IFNβ demonstrated robust tyrosine 701 phosphorylation of Stat-1 (p-Stat-1) within 10 min (Figure 1A). This phosphorylation was sustained for 30 min in IFNβ, not IFNα, stimulated cultures. Basal levels of p-Stat-1 were undetectable in these cultures. Western blot analysis for tyrosine 705 phosphorylation of Stat-3 (p-Stat-3) displayed a nine-fold increase in IFNβ stimulated cells however this was not replicated in IFNα cultures (Figure 1B). These data highlight the ability of type-1 IFNs to signal via the Jak-Stat pathway in this M17 cell line and identifies type-1 IFN subtype specificity in the phosphorylation of this pathway. Specifically, IFNα preferentially induces p-Stat-1 opposed to Stat-3, unlike IFNβ that is non-selective in its phosphorylation of the Stat isoforms analysed.

### Oxygen glucose deprivation initiates a neuro-inflammatory response in M17 cells

**Figure 2:**
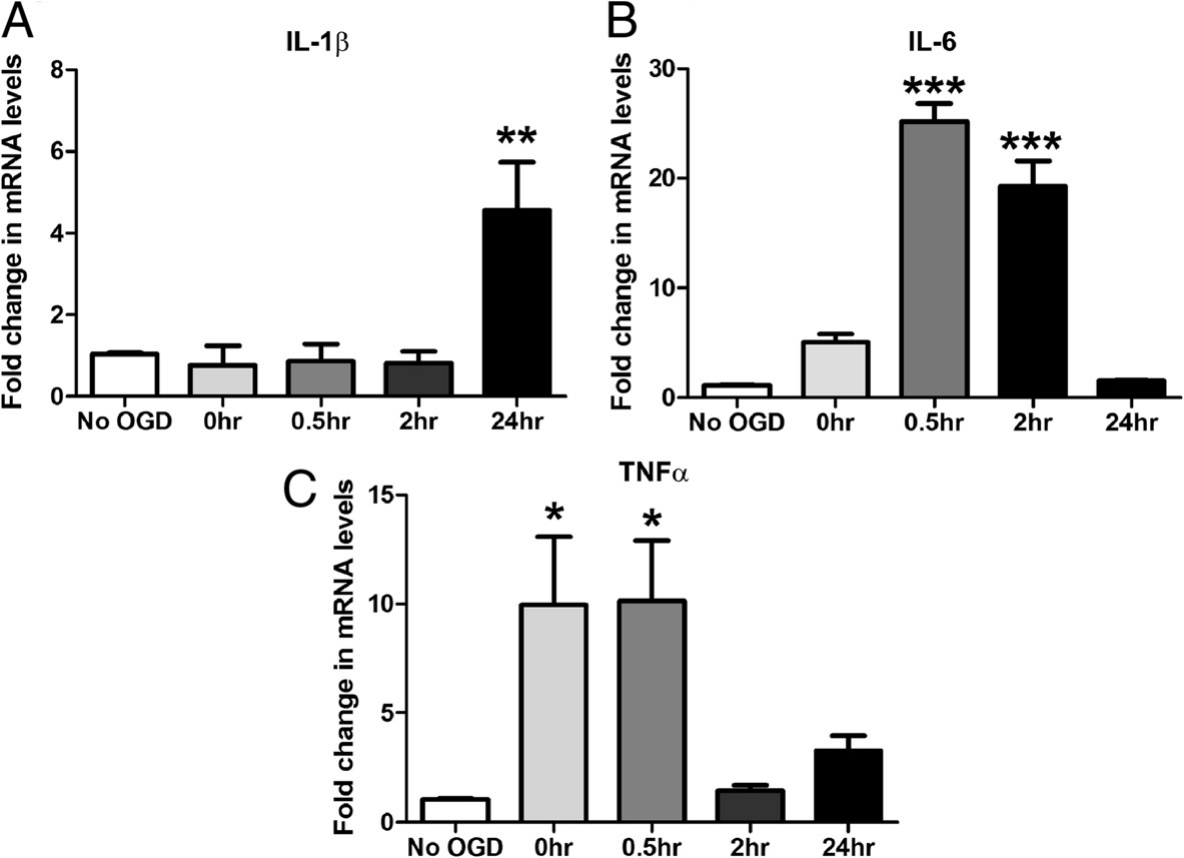
**Oxygen glucose deprivation (OGD) stimulates a classical innate inflammatory cascade involving an up regulation in IL-1β, IL-6 and TNF-α.** M17 cells were subjected to 4.5 h of OGD and reperfused for a 0 to 24 h period. Q-PCR was then performed detecting: **(A)** IL-1β, **(B)** IL-6 and **(C)** TNFα mRNA levels (*P <0.05, **P <0.01, ***P <0.001, n = 3, unmatched one-way ANOVA, Dunnett’s post-hoc test). Data are expressed as fold change in mRNA transcript levels in reference to no OGD control samples. Reperfusion times are indicated in hours on the x axis and graphical data are represented as mean ± SEM.

Mounting evidence suggests neuro-inflammation is a key contributor to the severity of CNS hypoxia-ischaemia injury. To model this hypoxia-ischaemia environment *in vitro* we utilised an oxygen glucose deprivation (OGD) model [34]. To characterise the pro-inflammatory response induced by this model we analysed cytokine expression by Q-PCR. Following reperfusion, M17 cells subjected to 4.5 h of OGD displayed a neuro-inflammatory response as shown by increased expression of hallmark pro-inflammatory cytokines. IL-1β transcripts were elevated 4.2-fold following OGD and 24 h reperfusion compared to control (Figure 2A). At 30 min and 2 h of reperfusion, IL-6 mRNA was upregulated 24-fold and 20-fold, respectively (Figure 2B). Finally, TNFα levels were upregulated 10-fold with 0 h of reperfusion and this elevation was maintained for 30 min (Figure 2C). Collectively this verifies a robust and reproducible neuro-inflammatory response in our model of hypoxia-ischaemia injury, involving critical pro-inflammatory cytokines known to be deleterious to injury outcome.

### Type-1 IFN production and signalling plays a role in the OGD response

**Figure 3:**
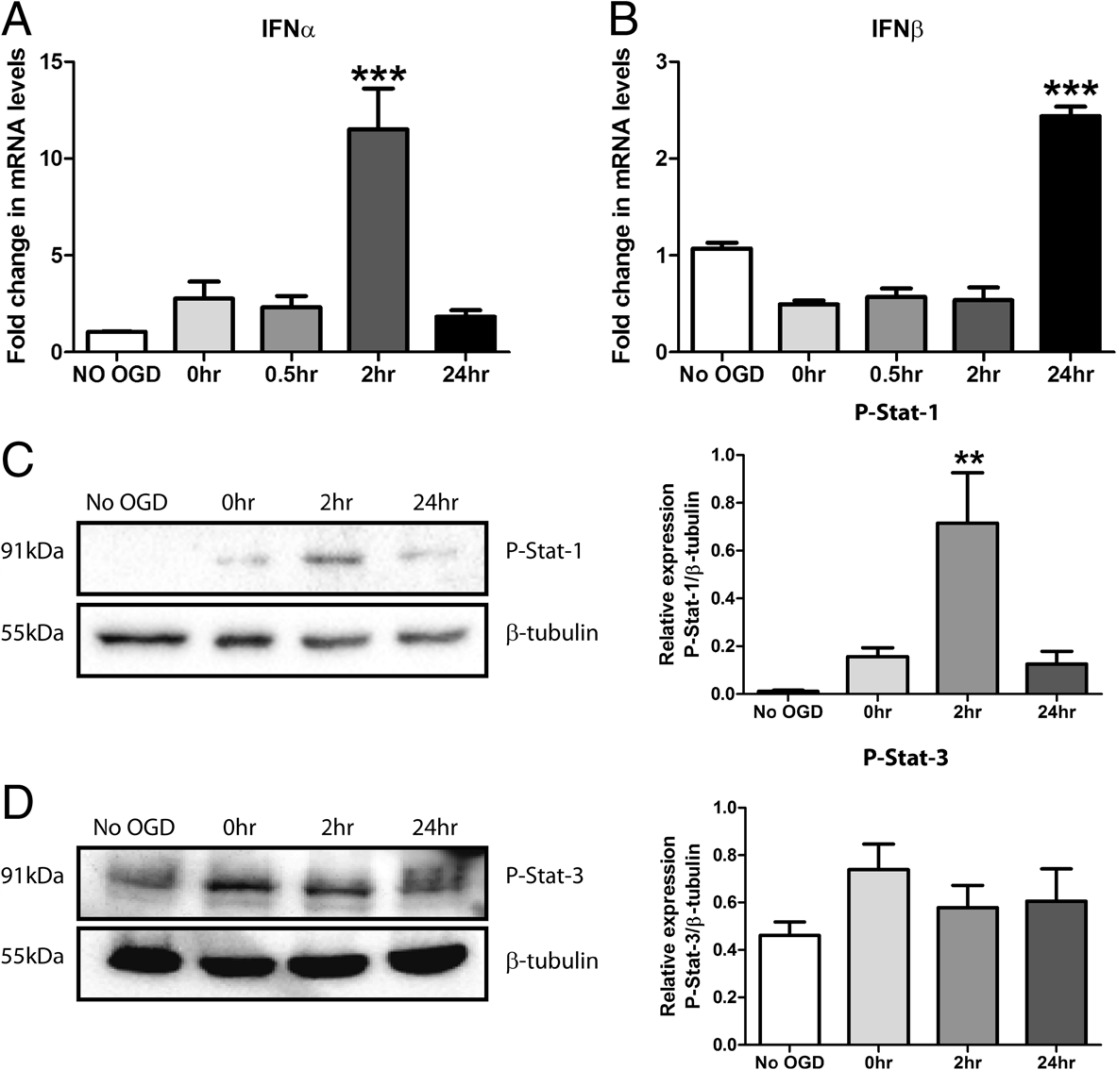
**Type-1 IFN production and signalling plays a role in the cellular response to OGD insult.** M17 cells were subjected to 4.5 h of OGD and reperfused for a 0 to 24 h period. Q-PCR was then performed detecting: **(A)** IFNα and **(B)** IFNβ mRNA levels (***P <0.001, n = 3, unmatched one-way ANOVA, Dunnett’s post-hoc test). Western blot analysis was performed on total cell extracts probing for **(C)** P-Stat-1 and **(D)** P-Stat-3 and corresponding densitometry is shown (**P <0.01, n = 4, unmatched one-way ANOVA, Dunnett’s post-hoc test). For densitometry calculations, phosphorylation intensity was measured in arbitrary units (A.U.) and normalised to the β-tubulin loading control. Graphical data are represented as mean ± SEM.

An upregulation of key interleukins has been confirmed in this model of OGD. The JAK-Stat signalling cascade is known to govern their production. Type-1 IFNs are key activators of this pathway and induce phosphorylation of Stat proteins in the M17 cell line (Figure 1). Thus to confirm the involvement of type-1 IFN signalling after OGD we investigated production and signalling of type-1 IFNs in response to OGD. Q-PCR showed a significant 11-fold upregulation in IFNα at 2 h reperfusion (Figure 3A) and 2.3-fold increase in IFNβ at 24 h reperfusion (Figure 3B). Western blot analysis demonstrated robust p-Stat-1 at 2 h reperfusion (Figure 3C), corresponding with the previous upregulation of IFNα at the same time-point. Conversely, p-Stat-3 levels remained unchanged across the reperfusion period (Figure 3D). These data support a novel role for type-1 IFN production and signalling in the neuro-inflammatory response to OGD *in vitro*. Moreover, IFNα signalling through Stat-1 appears to be an early activated pathway in this response.

### Removal of type-1 IFN signalling reduces pro-inflammatory cytokine load following OGD

**Figure 4:**
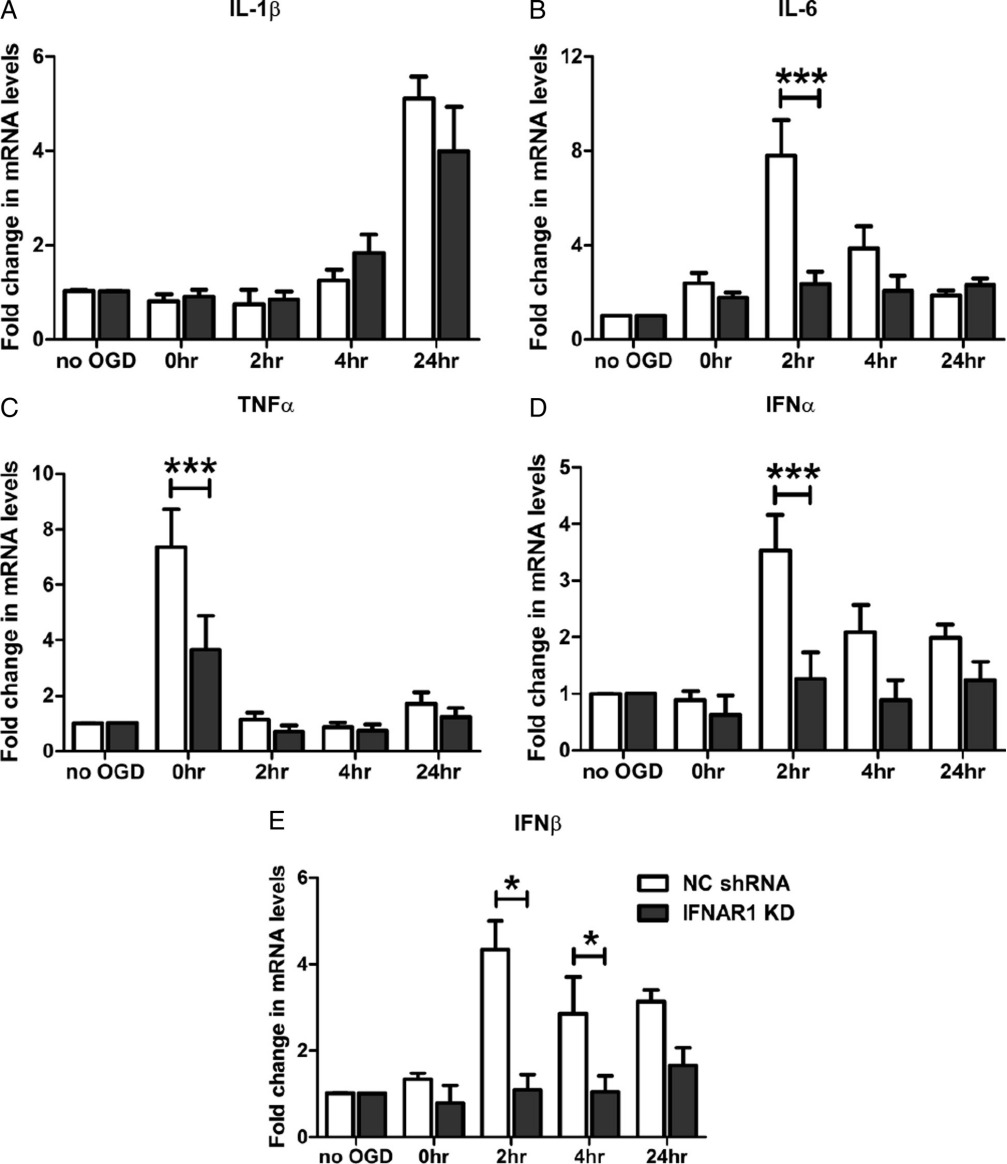
**Removal of type-1 IFN signalling confers a decreased pro-inflammatory response to OGD.** M17 NC shRNA and IFNAR1 KD cells were subjected to 4.5 h of OGD and up to 24 h of reperfusion. Q-PCR was then performed detecting: **(A)** IL-1β, **(B)** IL-6, **(C)** TNFα, **(D)** IFNα and **(E)** IFNβ mRNA levels (*P <0.05, ***P <0.001, n = 5, unmatched two-way ANOVA, Bonferroni post-hoc test). Data are expressed as fold change in mRNA transcript levels in reference to no OGD control samples. Reperfusion times are indicated in hours on the x axis and graphical data are represented as mean ± SEM.

Type-1 IFNs require both IFNAR1 and IFNAR2 subunits to activate downstream pro-inflammatory signalling cascades. In light of the identification that type-1 IFNs are novel ligands contributing to neuro-inflammation during hypoxia-ischaemia insult, we investigated the effect of removing type-1 IFN signalling through modulation of the IFNAR1 subunit. M17 cells stably transfected with either an IFNAR1 knockdown shRNA construct (IFNAR1 KD) or scrambled negative control construct (NC shRNA) were subjected to 4.5 h of OGD and 0 to 24 h of reperfusion. Q-PCR demonstrated no change in the IL-1β response between the two genotypes (Figure 4A). However, decreased IL-6 (9.7-fold *vs.* 2.9-fold, 2 h reperfusion, Figure 4B) and TNFα (8.3-fold *vs.* 4.5-fold, 0 h reperfusion, Figure 4C) mRNA transcript levels were identified in IFNAR1 KD M17 cells compared with their NC shRNA counterparts. The IFNAR1 subunit is critical in permitting autocrine production of the type-1 IFNs through the Jak-Stat signalling cascade. Considering this, we investigated type-1 IFN production in response to OGD in the absence of their cognate receptor subunit IFNAR1. Significantly decreased IFNα mRNA levels were observed at 2 h (3.5-fold *vs.* 1.3-fold) and, although not statistically significant, this trend was maintained until 4 h (2.1-fold *vs.* 0.9-fold) reperfusion in IFNAR1 KD cells compared to NC shRNA cells (Figure 4D). Significantly, IFNβ levels were decreased in IFNAR1 KD cells at 2 h (4.5-fold *vs.* 1.3-fold) and 4 h (3.7-fold *vs.* 1.4-fold) in comparison to NC shRNA cells (Figure 4E). These findings suggest that IL-6, TNFα, IFNα and IFNβ release in response to OGD is mediated by IFNAR1 and subsequent type-1 IFN signalling cascades.

**Figure 5:**
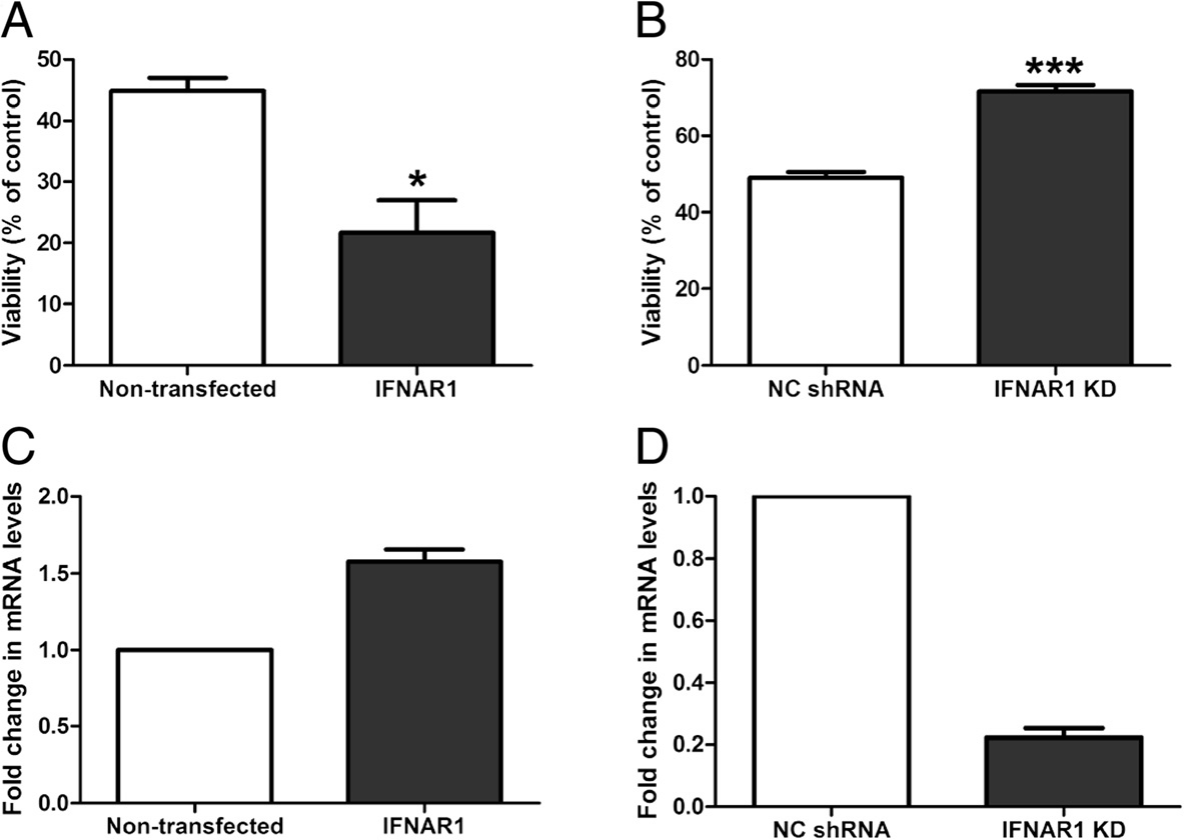
**IFNAR1 dependant type-1 IFN signalling is detrimental to cellular viability in response to OGD.** M17 cells either **(A)** over-expressing IFNAR1 or **(B)** with IFNAR1 knocked down were subjected to 4.5 h of OGD and 24 h of reperfusion and an MTT assay for cell viability was performed (*P <0.05, ***P <0.01, n = 4, unpaired two-tailed student’s *t*-test). Q-PCR was used to quantify IFNAR1 levels in M17 cells transiently transfected with **(C)** a pcDNA6.2/cEM-GFP-IFNAR1 over-expression plasmid or stably expressing **(D)** an IFNAR1 shRNA construct (n = 3). Graphical data are represented as mean ± SEM.

### Type-1 IFN signalling is deleterious in OGD and ablation of this signalling confers cellular protection

Neuro-inflammation is emerging as an important contributor to neuro-degeneration. The IFNAR1 KD M17 cells display decreased levels of pro-inflammatory cytokines in response to OGD compared to NC shRNA cells. We therefore assessed the effect of removing type-1 IFN signalling on cellular viability in response to OGD. The 4.5 h OGD treatment accompanied by 24 h of reperfusion routinely gives 40% to 50% cell death (quantified by MTT assay previously) in the M17 cell cultures. Our laboratory has utilised this method of OGD previously in primary cultured neurons and glia [34]. This longer period of OGD allows for a combinatorial effect whereby viable cells not only react to the OGD stimulus but also to progressive cell death during the reperfusion phase, modelling a penumbra-like environment. Cells transiently over expressing IFNAR1 display significantly decreased cell viability (44.9 ± 2.1% *vs.* 21.6 ± 5.3%) compared to non-transfected cells in response to OGD (Figure 5A). Furthermore stable knockdown of IFNAR1 confers significant protection following the same insult; 49.0 ± 1.5% *vs.* 71.6 ± 1.7% cell viability, negative control and IFNAR1 knockdown, respectively (Figure 5B). These viability alterations related to a 55% over-expression (Figure 5C) or 80% knockdown (Figure 5D) of IFNAR1 in the M17 cultures, as quantified by Q-PCR. These findings conclusively show that type-1 IFN signalling irrespective of ligand subtype is detrimental in hypoxia-ischaemia-induced neuro-inflammation. Specifically, the IFNAR1 subunit is critical in determining the cell viability outcome following OGD.

### Removal of the IFNAR1 subunit confers reductions in cleaved caspase-3 levels in response to OGD

**Figure 6:**
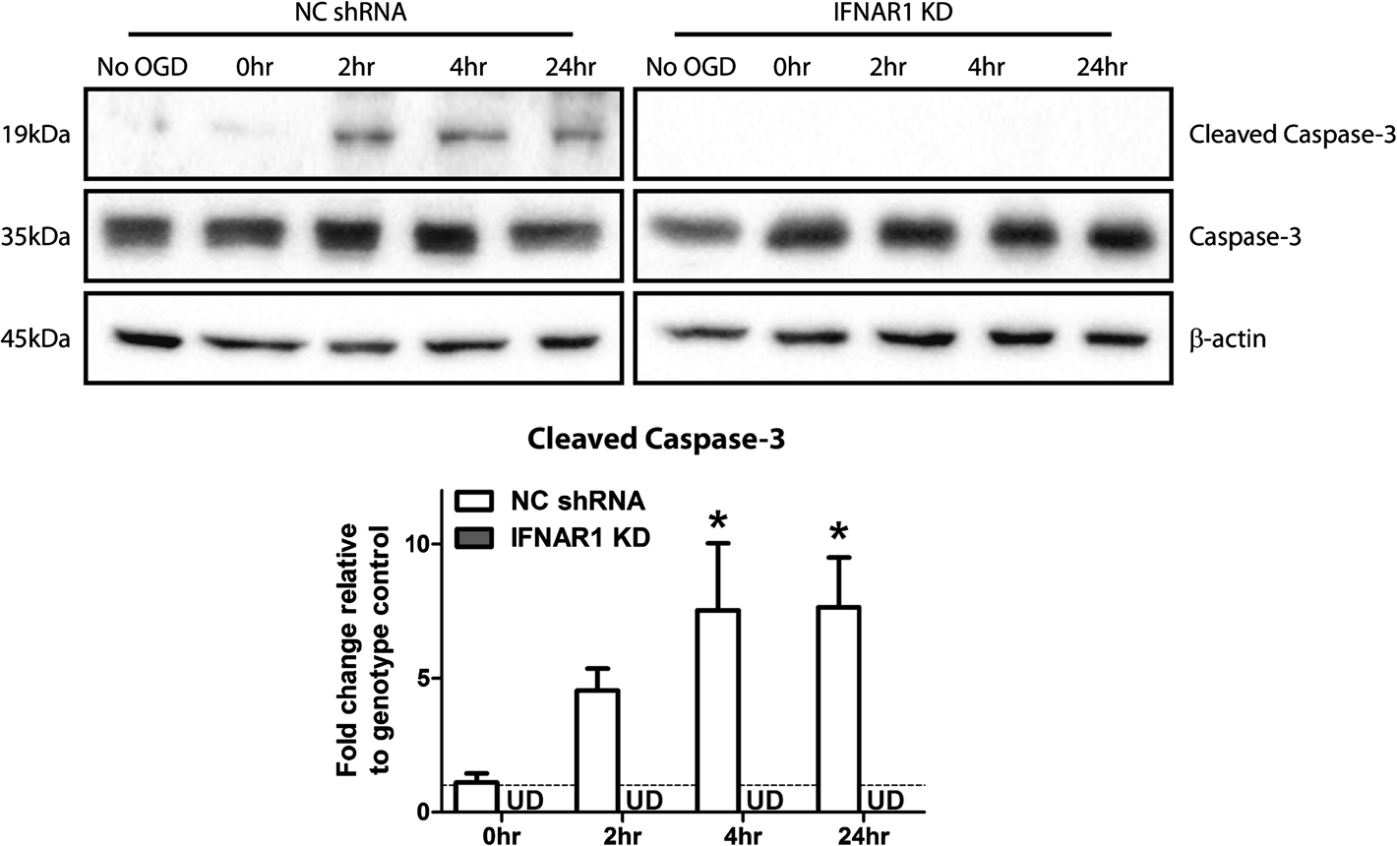
**Ablation of type-1 IFN signalling, through removal of IFNAR1, decreases pro-apoptotic cleaved-caspase-3 levels following O GD.** M17 NC shRNA and IFNAR1 KD cells were subjected to 4.5 h of OGD and up to 24 h of reperfusion. Cell lysates were analysed by western blot probed for cleaved and total caspase-3 levels (*P <0.05, n = 5, unmatched one-way ANOVA, Dunnett’s post-hoc test); β-actin was used as a loading control. For densitometry calculations cleaved caspase-3 intensity was measured in arbitrary units (A. U.) and normalised to the Caspase-3: β-actin intensity ratio. These values were then expressed as fold change to the relevant genotype control and are displayed as mean ± SEM. No statistical analysis was performed on the IFNAR1 KD blots as bands remained undetected across all time-points.

Within a neuro-inflammatory environment there are multiple cell death mechanisms activated which contribute to the overall tissue viability following injury. Necrosis, apoptosis (extrinsic and intrinsic variants) and necroptosis have all been reported in hypoxia-ischaemia injury [35]. Our data suggest a protective effect with the removal of type-1 IFN signalling, through IFNAR1 knockdown, in response to OGD. Therefore, we investigated the effects of removing IFNAR1 on ‘committed’ caspase-3 driven apoptosis in this environment. Western blot analysis for cleaved caspase-3 identified significantly elevated cleavage at 4 and 24 h reperfusion post OGD in NC shRNA but not in IFNAR1 KD cultures (Figure 6). This finding highlights that removal of type-1 IFN signalling is protective through mechanisms of reduced committed apoptosis in response to OGD.

## Discussion

Reducing the cellular damage within the core infarct area following hypoxic-ischaemic brain injury is considered to be largely uncontrollable; however limiting the development of the penumbra and progression of this injury may be more achievable. Therapeutic intervention requires a greater understanding of the cellular processes occurring in the injured environment, which remain uncharacterised. Neuro-inflammatory cascades are known to be involved in the progression of hypoxic-ischaemic injury; however the cytokine profile and cell-specific responses have not been fully elucidated. Our data suggest a previously unrecognised role for type-1 IFN production and signalling in response to OGD insult. IFNα production and downstream pro-inflammatory Stat-1 phosphorylation were upregulated during early stage reperfusion *in vitro* following OGD treatment. This phenomenon occurred in a neuro-inflammatory environment where the hallmark pro-inflammatory cytokines, IL-1β, IL-6 and TNFα, are readily secreted. Removing type-1 IFN signalling in the IFNAR1 KD cells decreased pro-inflammatory cytokine release and type-1 IFN levels in response to OGD. These same cultures were protected from the hypoxic-ischaemic insult and displayed reduced caspase-3 cleavage. This response was reversed when IFNAR1 over-expressing cells, promoting type-1 IFN signalling, were exposed to the same conditions.

Type-1 IFNs have well characterised pro-inflammatory and anti-viral roles in peripheral physiology but remain largely uncharacterised in the CNS. Neurons and glia are known to produce and respond to type-1 IFNs, however their role in neuropathologies is still largely unknown. Among a myriad of signalling cascades, type-1 IFNs can activate the Jak/Stat pathway and induce pro-inflammatory gene transcription, however much about ligand specificity and subsequent Stat isoform phosphorylation in CNS cell types is still not known. This current study identified an ability of type-1 IFNs to induce differential phosphorylation of Stat isoforms based on the initial IFN subtype. IFNα displays preference to activating Stat-1 as opposed to Stat-3, while IFNβ stimulation of the M17 cultures induced phosphorylation of both isoforms. This finding gives insight into type-1 IFN ligand specificity in a CNS-derived cell-line and supports previous findings that downstream type-1 IFN cascades are selectively activated based on ligand subtype [36,37]. At this time, 13 subtypes of IFNα alone have been discovered, with a universal type-1 IFNα used in this study. Therefore, further characterisation of individual IFNα subtypes is required.

Microglia are the primary immune cell of the CNS and play an important role in the final outcome of a hypoxic-ischaemic injury, however it is equally important to characterise the contribution and response of neuronal cells in this environment. Resident CNS cells secrete inflammatory cytokines IL-6 [12,38], IL-17 [39] and TNFα [13] within the developing penumbra area. The M17 neuroblastoma cells used in the current study have been reported to initiate a neuro-inflammatory response upon stimulation with the inflammatory and neurotoxic peptide amyloid-β (Aβ) 1-42 [40]. This pro-inflammatory response involved the secretion of type-1 IFN, IL-1β and IL-6. The M17 cell inflammatory response and Aβ1-42 cytotoxicity profile resembled that of primary murine cortical and hippocampal neurons used in the same study. Mechanisms of TNF-α-induced neurotoxicity have been repeatedly investigated using the closely related SH-SY5Y human neuroblastoma cell line. TNF-α treatment of these cultures induces apoptosis [41,42], as reported in primary neuronal cultures, with phosphorylation of NF-κB p65 and Stat proteins being implicated in the toxicity mechanism [43]. Knockdown of TLR8, upstream of these pro-inflammatory molecules, in SH-SY5Y cultures confers protection against OGD [44]. In the current study, M17 neuroblastoma cultures subjected to OGD demonstrated elevated expression of interleukin and TNF-α during the reperfusion phase. These findings confirm that OGD invokes a pro-inflammatory stimulus within the culture environment. More importantly, the timing of the cytokine response implies that it is occurring as a result of the core damage during the OGD period. We hypothesise that neuro-inflammation exacerbates the secondary injury and progressive cell death, playing a pivotal role in the reperfusion setting.

During periods of ischaemia, tissue undergoes necrosis [45] and releases cellular debris, detected by TLRs on microglia, astrocytes and neurons themselves. Ligand docking to TLR4 leads to direct activation of AP1 and NFκB transcription factors, leading to cytokine secretion [46], cellular infiltration and subsequent apoptosis [47]. The type-1 IFNs have been considered the master regulator of the innate immune systems cytokine response signature and hence control the initiation of the inflammatory response [19]. Taking this into consideration, the timing of a type-1 IFN response is crucial in the inflammatory response to hypoxic-ischaemic insult. In this study we demonstrate early upregulation of IFNα during the reperfusion period and this correlates with increased Stat-1 phosphorylation at the same 2-h time-point. This promotes IFNα as a novel CNS cytokine contributing to the neuro-inflammatory environment stimulated by OGD. Furthermore the timing of the IFNα response occurs at the beginning of the inflammatory event, highlighting the potential to modulate the developing inflammation. The current study demonstrated an IFNα preferential phosphorylation of Stat-1 in M17 cells. We propose that OGD stimulates IFNα release, selectively phosphorylating Stat-1 and contributes to a deleterious neuro-inflammatory cycle. Utilising the M17 neuroblastoma cultures, of human origin, gives insight into cellular inflammatory mechanisms behind the human brain response to hypoxia-ischaemia. Despite this characteristic they are indeed oncogenic and the possibility of an altered type-1 IFN inflammasome, compared to human cortical neurons, must be taken into consideration. Basal levels of p-Stat-3 were detected in the M17 cell cultures with no OGD treatment which may have masked potential upregulations of this mediator in response to OGD. Stat-3 is involved in the regulation of cell cycle [48] and may be altered in the proliferative M17 cell cultures. Furthermore other interleukins, namely IL-6, may also engender direct Jak/Stat activation through gp130 [49] and contribute to neuro-inflammation, however our data support a novel role for IFNα in perpetuating this neuro-inflammatory response. This finding has important implications as the type-1 IFNs are known to cross-talk with NFκB through regulation of interferon regulatory factor (IRF) 7 expression [50]. Considering the importance of NFκB in generating a neuro-degenerative cytokine ‘storm’, the type-1 IFN signalling cascades may provide novel avenues for therapeutic development.

Type-1 IFNs require their cognate receptor (interferon alpha receptor (IFNAR)), comprised of IFNAR1 and IFNAR2 subunits to bind and signal via the Jak/Stat cascade [18]. Furthermore, studies suggest that the IFNAR1 subunit is responsible for type-1 IFN subtype recognition and subsequent differential signalling [51,52]. Over-expression of the IFNAR1 subunit, increasing type-1 IFN signal transduction, resulted in M17 cells being more susceptible to OGD induced death. In contrast, knockdown of the IFNAR1 subunit reversed this detrimental phenotype with reduced pro-inflammatory cytokines and subsequent neuro-protection. Uncontrolled neuro-inflammation is able to facilitate cell death through multiple degenerative mechanisms including extrinsic and intrinsic apoptosis. Ablation of type-1 IFN signalling through removal of IFNAR1 resulted in reduced levels of pro-apoptotic cleaved caspase-3 in the OGD environment. These data suggest that removal of IFNAR1 confers protection through limiting apoptosis, pertinent as caspase-3 pathway inhibitors have been previously protective in stroke outcome [53]. Thus, we propose that type-1 IFN signalling is detrimental in hypoxic-ischaemic injury and modulation of this signalling may be beneficial to injury outcome. This study demonstrates the net effect of removing type-1 IFN signalling, however considering the pleiotropic nature [54,55] of the type-1 IFNs and their potential for beneficial functions in neuro-inflammation, a subtype specific functional analysis should be considered. Furthermore, this study investigates knockdown of IFNAR1 in M17 neuroblastoma cells alone in the context of OGD. While this approach identifies a critical neuronal type-1 IFN response in OGD-induced neuro-degeneration, these cells are normally embedded in a complex matrix of glial cells and form a cohesive system. It will be intriguing to identify if this protective phenotype is maintained in the brain environment where the presence of astrocytes and microglia enhance the severity of the cytokine storm in conditions of hypoxia-ischaemia and, if type-1 IFN signalling is critical in all cell types or just neurons.

In light of the pleiotropic nature of the type-1 IFNs, targeting IFNAR1 in the CNS therapeutically should be addressed with caution. The brain displays a unique IFNAR expression profile whereby IFNAR1 is readily expressed [56] but the IFNAR2 subunit is scarce (http://www.brain-map.org), indicating that peripheral type-1 IFN signalling differs from the CNS. Indeed IFNβ can signal independent of IFNAR2 [57], which considering the known receptor subunit imbalance, is crucial to potential signalling in the brain. These observations highlight that targeting IFNAR1 alone is a useful tool in controlling type-1 IFN signalling in the brain and we hypothesise that therapeutic modulation of this receptor shall prove beneficial in hypoxia-ischaemia injury outcome. However type-1 IFN signalling is crucial in the regulation of innate immunity, critical in viral immunity, beneficial inflammatory responses and protection from autoimmune disease such as experimental autoimmune encephalomyelitis [58]. In conditions of brain hypoxia-ischaemia, activation of innate immune system is required for microglia to successfully remove cellular debris from the inflamed environment and this process is largely considered protective. Inhibiting IFNAR1-dependent signalling may induce central immuno-suppression, which renders this reparative clearance mechanism inactive, perpetuating tissue damage and increasing penumbra size. It is clear that timing, degree of modulation, and, cell-type specificity are key factors in determining the potential therapeutic benefit of modulating IFNAR1. This study proposes that modulation of neuronal IFNAR1 levels or activity may be beneficial in controlling cellular damage following hypoxia-ischaemia injuries.

Neuro-inflammation is a double-edged sword. A delicate balance exists between a protective clearance role, where inflammation is resolved, and a deleterious role where unresolved inflammatory processes drive cell death. We hypothesise that type-1 IFN signalling is a key process in controlling this neuro-inflammatory environment and contributes to the deleterious weight of the neuro-inflammatory fulcrum. We further propose that modulating type-1 IFN production and/or signalling in the CNS may enable beneficial immune-modulation and improve physiological outcome. Clearly a greater body of knowledge of the neuro-inflammatory cascades governing neuro-degeneration in hypoxic-ischaemic injuries is required; however our study supports targeting type-1 IFN signalling as a novel therapeutic strategy.

## Abbreviations

Aβ: Amyloid-β; AP: Activator protein; CCL: Chemokine (C-C motif) ligand; CNS: Central nervous system; DAMP: Damage associated molecular pattern; gp: Glycoprotein; IFN: Interferon; IFNAR: Interferon alpha receptor; IL: Interleukin; IRF: Interferon regulatory factor; JAK: Janus associated kinase; NF-κB: Nuclear factor kappa B; OGD: Oxygen glucose deprivation; STAT: Signal transducer and activator of transcription; TLR: Toll-like receptor; TNF: Tumour necrosis factor.

## Competing interests

The authors declare that they have no competing interests.

## Authors’ contributions

MRM, MZ and RCA conducted all experiments and analysed the data. MRM, PJC and JMT designed the study and wrote the manuscript. PJC and JMT also contributed to all data analysis. All authors discussed results and commented on the manuscript. All authors read and approved the final manuscript.
